# Supplementary figures and images for: Piloting of a minimum data set for older people living in care homes in England: protocol for a longitudinal, mixed-methods study
Source: BMJ Open. 2023 Feb 27;13(2):e071686. doi: 10.1136/bmjopen-2023-071686 (PMC9972423; doi:10.1136/bmjopen-2023-071686)

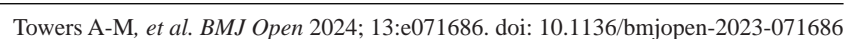

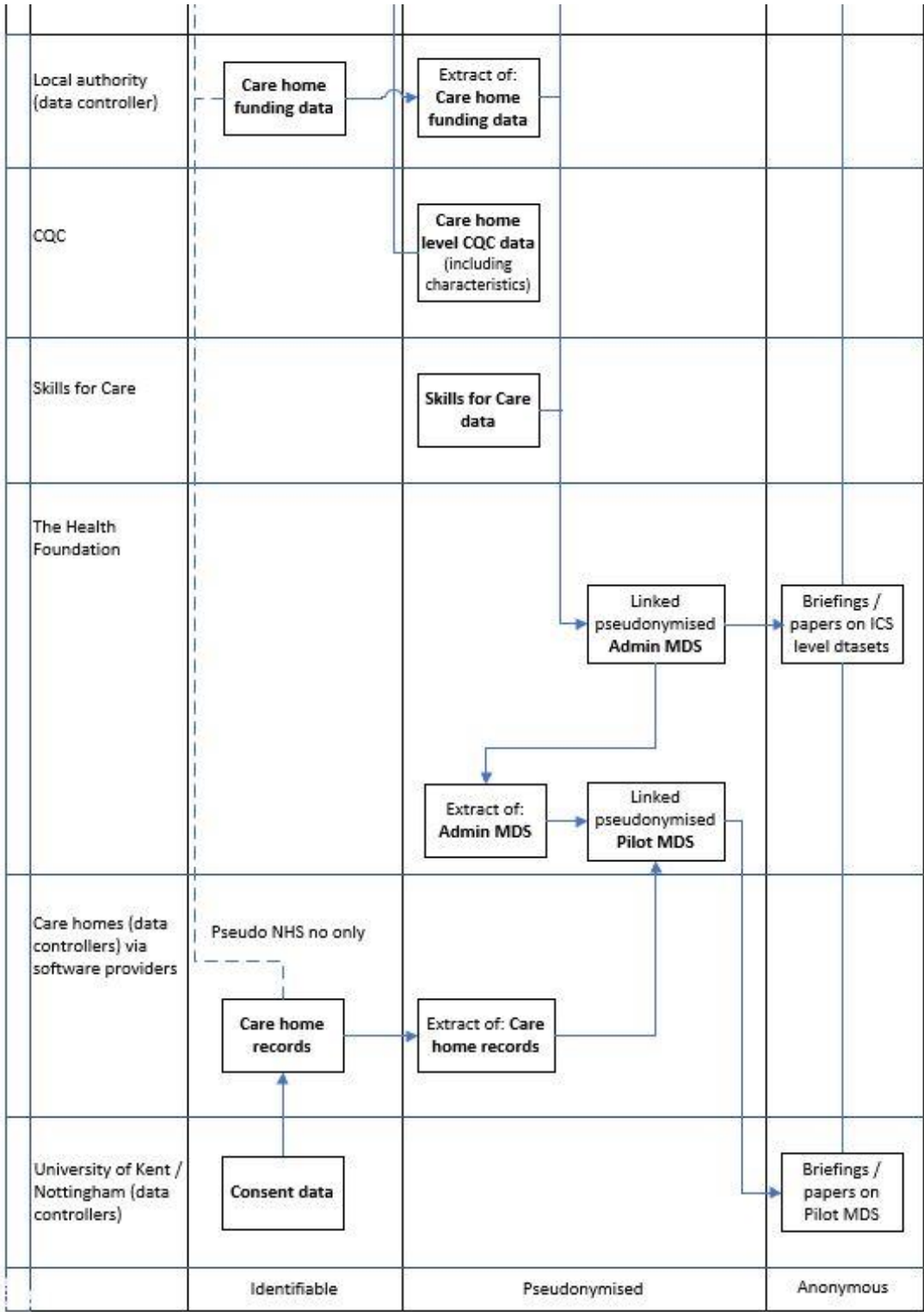

Supplement: Supplementary data [file bmjopen-2023-071686supp001.pdf]
