# Supplementary material for: Piloting of a minimum data set for older people living in care homes in England: protocol for a longitudinal, mixed-methods study
Source: BMJ Open. 2023 Feb 27;13(2):e071686. doi: 10.1136/bmjopen-2023-071686 (PMC9972423; doi:10.1136/bmjopen-2023-071686)
Supplement: Author's manuscript [file bmjopen-2023-071686.draft_revisions.pdf]

BMJ Open

BMJ Open is committed to open peer review. As part of this commitment we make the peer review history of every article we publish publicly available.

When an article is published we post the peer reviewers' comments and the authors' responses online. We also post the versions of the paper that were used during peer review. These are the versions that the peer review comments apply to.

The versions of the paper that follow are the versions that were submitted during the peer review process. They are not the versions of record or the final published versions. They should not be cited or distributed as the published version of this manuscript.

BMJ Open is an open access journal and the full, final, typeset and author-corrected version of record of the manuscript is available on our site with no access controls, subscription charges or pay-per-view fees (http://bmjopen.bmj.com).

If you have any questions on BMJ Open's open peer review process please email info.bmjopen@bmj.com

BMJ Open

A longitudinal, mixed-methods, pilot of a minimum data set (MDS) for older people living in care homes in England: study protocol.

Journal:	*BMJ Open*
Manuscript ID	bmjopen-2023-071686
Article Type:	Protocol
Date Submitted by the Author:	13-Jan-2023
Complete List of Authors:	Towers, Ann-Marie; University of Kent, Centre for Health Services Studies; NIHR Applied Research Collaboration Kent, Surrey and Sussex<br>Gordon, Adam; University of Nottingham, School of Medicine; NIHR Applied Research Collaboration East Midlands<br>Wolters, Arne; The Health Foundation, Data Analytics<br>Allan, Stephen; University of Kent, Personal Social Services Research Unit<br>Rand, Stacey; University of Kent, Personal Social Services Research Unit<br>Webster, Lucy; University of Kent, Centre for Health Services Studies<br>Crellin, Elizabeth; The Health Foundation, Improvement Analytics Unit<br>Brine, Richard; Health Foundation, Data Analytics<br>De Corte, Kaat; The Health Foundation, Data Analytics<br>Akdur, Gizdem; University of Hertfordshire, Centre for Research in Public health and Community Care<br>Irvine, Lisa; University of Hertfordshire, Centre for Research in Public health and Community Care (CRIPACC)<br>Burton, Jennifer ; University of Glasgow, Institute of Cardiovascular and Medical Sciences<br>Hanratty, Barbara; Newcastle University, Institute of Health and Society / Newcastle University Institute for Ageing; NIHR Applied Research Collaboration North East and North Cumbria<br>Killett, Anne; Univ East Anglia, School of Health Sciences; NIHR Applied Research Collaboration East of England<br>Meyer, Julienne; City University London, School of Health Sciences; National Care Forum<br>Jones, Liz; National Care Forum<br>Goodman, Claire; University of Hertfordshire, Centre for Research in Public Health and Community Care; NIHR Applied Research Collaboration East of England
Keywords:	Protocols & guidelines < HEALTH SERVICES ADMINISTRATION & MANAGEMENT, Quality in health care < HEALTH SERVICES ADMINISTRATION & MANAGEMENT, GERIATRIC MEDICINE

A longitudinal, mixed-methods, pilot of a minimum data set (MDS) for older people living in care homes in England: study protocol.

Abstract

Introduction

Health and care data is routinely collected about care home residents in England, yet there is no way to collate these data to inform benchmarking and improvement. The Developing research resources And minimum data set for Care Homes' Adoption and use (DACHA) study has developed a prototype minimum data set (MDS) for piloting.

Methods and analysis

A mixed-methods longitudinal pilot study will be conducted in 60 care homes (approximately 960 residents) in three regions of England, using resident data from cloud-based digital care home records at two-time points. These will be linked to resident and care home level data held within routine NHS and social care data sets. Focus groups and individual interviews with care home staff, and external stakeholders will explore implementation and the perceived utility of the MDS. Data will be assessed for completeness and timeliness of completion. Descriptive statistics, including percentage floor and ceiling effects will establish data quality. For validated scales, construct validity will be assessed by hypothesis testing and exploratory factor analysis will establish structural validity. Internal consistency will be established using Cronbach's alpha. Longitudinal analysis of the pilot data will demonstrate the value of the MDS to each region. Qualitative data will be analysed inductively using thematic analysis to understand the complexities of implementing an MDS in care homes for older people.

Ethics and dissemination

The study has received ethical approval from the London Queen's Square Research Ethics Committee [22/LO/0250]. Findings will be disseminated to: academics working on data use and integration in long term care, care sector organisations, health and social care policy makers, and commissioners. Findings will be published as part of the National Institute of Health and Care Research Journal Series. Partner NIHR Applied Research Collaborations, the National Care Forum, and the British Geriatrics Society, will disseminate policy briefs.

Introduction

Approximately 420,000 older people live in care homes in England and Wales. Just under half of residents are admitted to care homes following hospital admission [1]. Older care home residents live with high levels of dependency, cognitive impairment and multi-morbidity [2]. Around 70% have dementia, though some will not have a formal diagnosis [3].

For peer review only - http://bmjopen.bmj.com/site/about/guidelines.xhtml

Data are routinely collected and held about residents by care homes and external agencies (e.g. NHS, LAs, the regulator). Some of this information is returned to different regulators, commissioners and policymakers for aggregated use. The lack of harmonisation of indicators means duplication of effort because similar information about residents is recorded in different ways for different uses [4]. Heterogeneity in the frequency of measurement, tools and measures deployed and the mode of recording [5], makes it difficult to compare or collate data at an aggregate level. Recent estimates suggest that approximately 30-40% of social care providers in England are still entirely paper-based, with another third only partially digital [6].

The pandemic highlighted the need to collate real-time information about residents' health and care needs and accelerated the move to digital data solutions through emergency legislation [6,7]. Tools, such as the Capacity Tracker in England, originally designed to manage hospital discharges, were repurposed to monitor infection and vaccination uptake rates, and collect workforce data. The new white paper, People at the Heart of Care, aims to reverse historical under-investment in adult social care technology [8]. The Government target is for 80% of care providers (including care homes) to be using digital care records by March 2024 [6]. As well as improving the accessibility and interoperability of routinely collected data in care homes, this presents opportunities to standardise the way data is recorded about residents' needs and outcomes for the purposes of an MDS.

Structured and standardised approaches to data in long-term care are well established in some countries. The Minimum Data Set (MDS 3.0) [9] in the United States is mandated for all nursing home residents funded by Medicare or Medicaid. The International Resident Assessment Instrument (interRAI) [10], initially derived from the MDS, is used in multiple countries including Canada and New Zealand, France, Belgium, Netherlands, Australia and Switzerland. These datasets enable care coordination, governance and audit, remuneration for services, service design and planning, and policy decisions [11]. Previous efforts to introduce interRAI in the UK have been unsuccessful [12,13], possibly because data capture was not incorporated into routine practice and was perceived as an additional administrative burden [4].

The DACHA study [14] is a programme of research (2019-2024) that aims to develop and test an MDS for care homes providing care to older people in England. There are five work packages (WPs), three of which are complete: systematic evidence reviews to inform measures and outcomes for inclusion in an MDS (WP1) [15]; creation of an individual patient data repository of UK care home trials (WP2) [16]; and realist review identifying programme theories describing what is involved when developing and implementing an MDS in care homes (WP3) [17].

Adopting an approach used in previous work [18], WP4, led by the Health Foundation, will produce a resident-level MDS from data *routinely collected* by health and social care providers. These data will not come directly from care homes, although homes will have

For peer review only - http://bmjopen.bmj.com/site/about/guidelines.xhtml

populated some of it for other purposes (e.g. for the Care Quality Commission (CQC)). Care home residents will be identified based on the address registered with their General Practitioner [18]. This protocol is for WP5, in which we augment WP4 data, to form a complete MDS, using data extracted from care homes' digital care records.

Methods and analysis

Aim and objectives

Aim

To undertake a longitudinal pilot of a prototype MDS for older people living in care homes using digital care records in England.

Objectives

1. Assess the feasibility of extracting data directly from digital care records and matching this to routinely collected health and social care data to populate a complete MDS.
2. Assess the quality of the complete MDS data.
3. Evaluate the usefulness of the MDS to stakeholders (health and social care, local authorities, care providers, residents and their families).
4. Assess potential barriers and facilitators to wider implementation of the MDS.

Study design

A mixed-methods longitudinal pilot of the prototype MDS will be conducted in 60 care homes for older people (nursing and residential) using digital care records from participating software vendors in three sites (n=20 homes per site)[1] and linking this data to routinely held health and social care data (see Table 1 for data sources). Each site will comprise an Integrated Care System (ICS). An ICS is a regional partnership between NHS organisations, local councils and other organisations including the third sector and social enterprises, to coordinate and provide health and social care. There are 42 ICSs across England. The study has chosen three ICSs (Nottingham and Nottinghamshire, Surrey Heartlands, and North East and North Cumbria), with a history of working collaboratively with care homes to improve care delivery.

Using a longitudinal design will enable us to assess the feasibility of the MDS by looking at completion rates and implementation over time.

[1] Care homes for people with intellectual and developmental disabilities will not be included in this study.

For peer review only - http://bmjopen.bmj.com/site/about/guidelines.xhtml

The prototype MDS

Given government policy to move rapidly to digital shared care records [6], we will work with homes that already have digital care software to plan and record residents' care. The final MDS, consisting of routinely-held health and social care data and data extracted from care records, will include information about residents' demographics, date of admission, health and care needs (including palliative care), quality of life, complications and adverse events, diagnoses, medication, vaccinations, health care use, care home characteristics, and workforce data (see Table 1 for data sources).

We are working with the Care Software Providers Association (CASPA), a consortium of software providers (*https://caspa.care*), and two software companies (Person Centred Software (*https://personcentredsoftware.com/gb/*) and Nourish (*https://nourishcare.co.uk/*). We have mapped the DACHA MDS variables against the data routinely collected in their systems and are working with them to address any gaps. For example, previous work identified that different stakeholders wanted information about residents' quality of life (QoL), yet this is not currently routinely collected [4,17]. QoL measures are therefore being added to the software for the pilot and will be available to care homes to inform direct care. We are also working with providers to add other items (e.g. cognitive impairment and activities of daily living) in a standardised format. Currently, descriptive care notes capture important information for direct care (e.g. about functioning) but not in a format suitable for quantitative analysis.

Sampling

Homes

We aim to recruit 20 care homes for older people in three ICSs (total N=60 homes) geographically dispersed across England (South East, Midlands and North East). The three ICSs have 279 care homes (11,121 beds), 222 homes (8,451 beds), and 636 homes (26,968 beds) respectively. Assuming an occupancy rate of 90% [1], the sample size required to give a true representation of the finite older care home population in each of the three ICSs, with 90% confidence and a 5% margin of error, is 262-268 residents [19].

Care homes will be eligible for inclusion if they are routinely using digital care records from the two participating software vendors. Based on figures provided by the software vendors, the eligible sample across the three respective sites for this study are 52 homes (3,093 beds), 51 homes (2,809 beds), and 66 homes (3,288 beds). To achieve an adequate resident sample for the study, we will stratify the eligible care homes by size, maintaining an even split of homes with and without on-site nursing (nursing and residential homes) in each ICS, where possible. We anticipate that approximately three out of every four care homes selected will be above average size (40 beds).

For peer review only - http://bmjopen.bmj.com/site/about/guidelines.xhtml

Based on earlier work and cognisant of ongoing post pandemic pressures we assume a conservative 33% response rate for residents [22] . Given the stratifying of eligible care homes, anticipated response rate and assuming 90% occupancy this would provide a sample of 358, 320 and 292 residents in each of the three ICSs on average (n=970 residents in total). Median length of stay for residents in care homes will vary by location and provider [18,23,24]. We will liaise with care home managers to assess any potential issue with occupancy rates and sample attrition (particularly recognising the smaller sample size in ICS three), looking to identify ways to resolve this, for example by setting higher recruitment targets for specific homes.

Residents

Inclusion and exclusion criteria

We will utilise a census approach where all permanent residents will be eligible to participate, including those lacking capacity to consent. To minimise research burden on potential participants going through acute transitions, and who may also move through the care home rapidly, we will exclude residents receiving respite or temporary/short-stay care, or people identified by the care home staff as being in the last weeks of life at the point of study inception.

Recruitment

All residents' capacity to consent will be assessed by a research nurse or member of the research team. We will support the recruitment of adults without mental capacity by using a personal consultee for those with a relative to act on their behalf, and a professional or nominated consultee for residents with nobody to act for them. We have used this approach in previous studies [12,25,26], in line with the Mental Capacity Act [27]. Consultee discussions may take place on the telephone or using videoconferencing technology.

Data collection from care homes

Resident data collected from care homes will be entirely extracted from the home's digital care records via the participating software vendors at baseline and six months. Participating care homes will be the data controllers. The software providers will be the data processors, meaning they will pseudonymise the data for participating residents (see data linkage below for details) and support the homes to transfer it securely to The Health Foundation. The pseudonymised data will be stored securely on The Health Foundation servers.

Data linkage and processing

Table 1 summarises the data to be collected and proposed data sources. We will use deterministic linkage to merge the data from digital care records with the routinely collected health and social care administrative data to complete the prototype MDS (see,

For peer review only - http://bmjopen.bmj.com/site/about/guidelines.xhtml

Data Flow Diagram in Supplementary information). This will include data on: demographics and care home stay [source – Personal Demographics Service] [28]; adverse events, diagnoses, medications and health care utilisation (e.g. primary care and hospital appointments, ambulance call outs) [sources - GP, Secondary Uses Service, NHS111 data sets]; care home characteristics [sources – CQC, local authority data sets]; and basic workforce data [source – Skills for Care] [29]. We will identify relevant participant identifiers, for example NHS number and/or National Insurance number, to complete this linkage. These identifiers will subsequently be pseudonymised using an agreed linkage key, allowing the data from digital care records to be linked to administrative data pseudonymised in the same way [30].

The linked dataset will be held by The Health Foundation in a dedicated secure data environment. The pseudonymisation key will be shared separately with the other data providers supplying administrative data for the purpose of the MDS. The Health Foundation staff will not have access to the pseudonymisation key but instead link the datasets together using pseudonymised identifiers, therefore making it unlikely that resident data can be re-identified.

All resident information will be processed in a dedicated secure environment that complies with national data security standards (NHS Digital Data Protection and Security Toolkit) and certified against an international standard for information security (ISO27001), access to this environment will be limited to trained analysts, and all analysis findings are subjected to rigorous statistical disclosure control procedures to make sure residents cannot be identified.

Care home level data

We will collect data on institutional variables for each care home to contextualise the evaluation and consider representativeness of the wider care home sector. To minimise research burden for care homes much of this data will be collected from the routinely held health and social care data described above. Data collected directly from care homes through a short online survey will include: number of beds; number of residents; proportion of self-funding residents; and the number of full-time equivalent care staff currently employed.

For peer review only - http://bmjopen.bmj.com/site/about/guidelines.xhtml

Table 1: Proposed data sources for the prototype MDS

	Sections	Example variables	Digital care records	Health and social care datasets
1	Demographics/ characteristics	Date of birth; sex; NHS number; area-based deprivation	No	Personal Demographics Service
		Religion, Languages, Marital or partnership status, Deprivation of liberty	Yes	No
		Ethnicity; weight; height	Yes	GP data; Secondary User Services data
2	Palliative Care Needs	End of life pathway register	No	GP data
3	Care Home Stay	Date of entry to care home; date of death	Yes	No
4	Resident Needs	Skin condition	Yes	No
		Cognitive impairment and impact on perception, understanding & need for support	Yes*	GP data; Secondary User Services data
		Oral/nutritional status	No	Secondary User Services data
		Continence	No	Community datasets (where available)
		Ability to perform activities of daily living; cognitive performance; delirium	Yes*	No
5	Quality of Life	Outcomes; mood; dementia quality of life	Yes*	No
6	Complications / Adverse Events	Infections	Yes	GP data; Secondary User Services data
		Falls (leading to hospital admission or GP visit)	Yes	Secondary User Services data; 999 data; Ambulance data

For peer review only - http://bmjopen.bmj.com/site/about/guidelines.xhtml

	Sections	Example variables	Digital care records	Health and social care datasets
		Falls (only captured at care home level); early warning score; unintended weight loss	Yes	No
7	Diagnoses	Past medical history	No	Secondary User Services data; GP data
		Frailty	No	GP data
		Adverse reactions and allergies	No	GP data
8	Medication and Vaccination	Prescribed medication and administered vaccines	No	GP data
9	Health Care Utilisation	Primary care use	No	GP data; NHS 111 data; 999 data
		Community nursing; Community allied health professionals	No	Community Services Data Set
		Out-of-hours contacts	No	GP out-of-hours data
		Ambulance call outs	No	Ambulance data
		A&E attendance; emergency admissions; secondary care usage (outpatient appointments & elective admission)	No	Secondary User Services data
10	Care Home Characteristics and workforce characteristics	Type of home; care home characteristics, specialities and client groups; location of care home; area-based deprivation; registered bed capacity; sector of provider; provider ownership type; CQC rating	No	CQC data
		Staffing model; staffing ratios; numbers and types of staff; number of agency staff; number and type of vacancies	No**	Skills for Care data

*Will be added to the software for the purposes of the pilot study

**As the Skills for Care workforce survey is voluntary, we will ask participating homes to provide some information on workforce as part of a short online survey for the pilot.

For peer review only

Feedback to care homes

We will give participating homes aggregated summaries of their own MDS data, benchmarked against other data from the whole sample in their ICS. This will include indicators of residents' health, use of services and social care outcomes (e.g. quality of life). We seek to understand the extent to which the MDS may generate useful information for care homes, esepcially in relation to service development and improvement.

Support for homes

To provide appropriate support to homes throughout, each ICS will have a dedicated, local researcher who will be their main point of contact for the study duration. Homes will be offered payment of £100 per home, per wave of data extraction (£200 in total), as a token of appreciation. Where possible, measures used in the pilot will be questionnaires developed for and/or used in care homes already and designed to be completed by staff. This should improve implementation and reduce burden on staff [15].

Focus groups and interviews

Care home managers and staff will be invited to participate in two rounds of focus group interviews (up to two hours long) with other care homes from their ICS or one-to-one interviews (up to an hour long) (see Table 2). These focus groups and interviews will explore:

- Implementation issues;
- Applicability and utility of the MDS for direct care;
- Potential modifications to the MDS.

Participation in these focus groups and interviews is voluntary, informed consent will be obtained and participants thanked for their time with a £20 gift voucher. All will be audio-recorded, with interviews facilitated by one researcher, and focus groups facilitated by two researchers. We will keep focus groups to a manageable size (up to 10 per group), running additional groups to accommodate views of staff from all participating homes if required.

At the end of the pilot, we will conduct telephone or face-to-face interviews with three key representatives from each ICS (n=9) to explore views of the implementation process and relevance of the data to their local priorities. To assess potential barriers and facilitators to the wider implementation of the MDS, the implementation aspects of the interviews and focus groups will be structured using the Consolidated Framework for Implementation Research (CFIR) [31].

A final stakeholder event will be held in each participating ICS area to present summary data on the MDS, constituent variables and linked health and social care outcomes. Group discussions will explore utility and feasibility of the MDS.

For peer review only - http://bmjopen.bmj.com/site/about/guidelines.xhtml

Table 2: Research activities by wave of data collection

	Data extraction and linkage	Aggregated feedback to homes	Focus groups and interviews
Wave 1	March 2023	May 2023	May-June 2023
Wave 2	September 2023	November 2023	November-Dec 2023

Data synthesis and analysis

This study will extract resident-level data from digital care records and pilot the process of matching this to routinely held health and social care data sources, collated by The Health Foundation. In addition, there will be data from focus groups and interviews. As such, there will be both quantitative and qualitative data collected by the study at different levels (resident, care home and ICS). We will use NVivo for windows [32] to store and analyse qualitative data. We will use R [33] and STATA [34] to analyse quantitative data.

Analysis of the implementation process

Analysis of the implementation process will be structured to test findings of earlier DACHA work describing what enables uptake and use of MDS in care home settings and what is perceived by different stakeholders as key to successful uptake (for example, shared aims, utility, ease of use) [15,17]. Data from each ICS will be analysed separately, to identify locality themes and then together to identify common themes.

Considering the psychometrics of the MDS

Descriptive statistics will be reported for MDS data, including the percentage floor (lowest score) and ceiling (highest score) for multi-item measures. Feasibility will be assessed by completeness (percentage missing data). To identify factors related to completion rates, bivariate analysis of the % completion rates for the MDS variables added for the pilot (e.g. QoL measures) and care home organisational factors (e.g. CQC rating, care home size, staffing level) will be conducted. For validated measures (e.g. quality of life), exploratory factor analysis will be used to assess the structural validity of the data in the MDS. Construct (convergent) validity will be assessed by hypothesis testing where there are either multiple measures of similar constructs, for example around Quality of Life, or we would expect to observe associations based on previously published research. Internal consistency of participants' responses will be described using Cronbach's alpha.

For peer review only - http://bmjopen.bmj.com/site/about/guidelines.xhtml

Analysing the ways in which MDS can be useful to key stakeholders

We will demonstrate the value of the MDS to stakeholders through analysis of the pilot data. Alongside The Health Foundation, we will introduce the prototype MDS to representatives of each ICS and seek to understand their local geography, service delivery and commissioning structures. Priority research and service development questions will be identified that can be addressed using the pilot data in an appropriate longitudinal study. For example, the MDS could be used to assess what drives hospital admissions from care homes. Sample size and power calculations will be considered during these discussions.

We have worked hard, through preparatory work in earlier work packages, to produce a parsimonious dataset that will minimise data collection burden on care homes. We will, though, at this point also explore variables, missing from the current MDS, which may improve its usefulness to key stakeholders.

Patient and Public Involvement

The DACHA study includes a programme of patient and public involvement (PPI) which holds regular meetings of patient and public involvement groups with relatives of people living in care homes, and care home staff in both direct care and management roles. Leadership of this group includes someone with experience both as a family carer and working in a care home. Care home residents are involved through collaboration with activity providers in their care homes. Stakeholders, including care providers, commissioners, quality monitoring and health providers are involved through structured national consultations. These stakeholders have shaped the earlier work packages, which have then fed into this MDS pilot. PPI representatives also sit on the study steering committee and attend team meetings.

For this pilot study, involvement has included consultation with PPI representatives from care homes, family carers and residents regarding the consent process, information sheets and consent forms, priorities for content of an MDS, quality of life measures included in the MDS, principles of data sharing and pseudonymization.

Input of PPI representatives will be sought throughout the study, commenting on findings from interviews and focus groups, and measurement issues raised by individual data items, or groups of items, within the dataset. We will also work with this group to develop Plain English dissemination materials.

Strengths and weaknesses

The care home sector is heterogeneous, and this is a pilot of an MDS in care homes using digital record software providers. This approach is in line with policy directives for 80% of providers to be using digital care records by March 2024. We are working with two software providers with the largest market coverage in England. Recruiting homes in three different regions of England, including one site with high social deprivation, will also maximise the

For peer review only - http://bmjopen.bmj.com/site/about/guidelines.xhtml

likelihood our cohort will sample a range of geographical and socio-demographic contexts. The sampling strategy described above aims to recruit a mixture of homes that are representative of the sector in terms of type (residential and nursing care), proprietorial status and size. Whilst we cannot ensure that the linked data will be nationally representative, we will be able to explore and report on the typicality of participating homes using national data held by the Health Foundation in WP4

All permanent residents will be eligible (except those known to be in the last weeks of life). This will enable us to attain a representative sample of care home residents for each ICS. Sampling could be affected by local variation in occupancy rates and length of stay, but we will be able to detect such variation using our data [18].

Our data linkage approach bringing together care home generated data with resident data held in routine datasets is innovative and addresses the need for integrated data. There are some risks. First, the NHS number used to link records could be recorded incorrectly, meaning the pseudo key will not match. We can mitigate against it by verifying NHS number in digital care home records, and measuring the extent to which it occurs. Second, residents consented in the pilot are not captured by the administrative dataset because their registered address has not been updated, or is not recognised as a care home by the Unique Property Reference Number (UPRN) algorithms used by The Health Foundation. We will mitigate against this by sharing the NHS Number directly from the providers to the NHS.

The dataset will be deployed in a real-world setting and then describe how organisational and individual stakeholder factors impact implementation using mixed methods. We will be able to understand the feasibility and usefulness of the dataset, refine its contents and establish how to implement it more widely at scale and pace. This work takes place against a backdrop of ongoing post pandemic service reconfiguration. We are mitigating against this by working with organisations active around data in care homes, including government agencies, to ensure our findings are cognisant of, and compatible with, other work underway or planned.

Ethics and Dissemination

The study has received a provisional favourable ethical approval from the London – Queen's Square Research Ethics Committee [22/LO/0250]. Residents and staff will all receive written and verbal information about the study from the research team or research nurses supporting the study. Where participants have capacity, informed consent will be gained. In accordance with the Mental Capacity Act [27], residents assessed as lacking the capacity to consent will be recruited via the advice of a personal or nominated consultee.

Findings will be disseminated to three key audiences: academics working on data use and integration in long term care, care sector representative organisations, and health and social care policy makers and commissioners. This will be through presentations at academic and clinical and sector specific conferences, webinars tailored for stakeholder groups, a report to the funder and publications in peer-reviewed journals. Findings will be summarised in plain English briefings and social media publications accessible to residents, relatives, and care

For peer review only - http://bmjopen.bmj.com/site/about/guidelines.xhtml

home staff. We will work with our partner NIHR Applied Research Collaborations, the National Care Forum colleagues, and the British Geriatrics Society, the national professional organisation representing health care professionals with an interest in the care of older people with frailty, to develop policy briefs aimed at commissioners and providers of health and social care.

The end product of this research will be a finalised digital MDS prototype. Although this will have been tested in English care homes, our aim is to develop a tool that will be useful across all four UK nations, with international relevance. This work will also create new knowledge on how to support care homes and other stakeholders to implement the prototype MDS, as well as the data linkage arrangements required. Ultimately, this will enable us to make recommendations that encourage widespread use of the MDS [4,6]. Piloting the MDS with homes already using digital care records will help to future-proof its use, and inform the Department of Health and Social Care's agenda for digitalisation of health and social care records [6].

Conclusion

This pilot will demonstrate the use and value of an MDS in care homes already using digital care planning software that is linked to resident data held routinely by local health and social care organisations. The rapid acceleration towards digital shared care records and the imminent establishment of a data framework for adult social care, underline the need for empirical evidence about what needs to be in place to implement these changes, whilst ensuring data are relevant to those providing, funding and regulating care for care home residents.

Supplementary Appendices

Data Flow Diagram: This is a flow diagram of the data being collected in the pilot study for the purposes of the MDS. It shows data sources and linkage.

Contributors: The study concept and design was conceived by CG, AMT, AG, AW, BH, JB, LI and LJ. SA, SR, RB, LC, KDC, LW and GA assisted in refining the design and creating tables and figures. AW, SA and SR designed the analysis plan, with input from the team. JM and AK led on PPI input. AMT led the drafting of the manuscript, with input from AG, AW, LW, SR and SA. All authors critically revised the manuscript and approve the submitted version

Competing interests: none declared

Funding: This work was funded by National Institute for Health Research (NIHR) Health Services and Delivery Research (HS&DR) Programme, grant number NIHR127234. Adam Gordon, Claire Goodman, Anne Killett, Barbara Hanratty and Ann-Marie Towers are part-funded by the NIHR Applied Research Collaborations for East Midlands (ARC-EM), East of England (ARC-EoE), North East and North Cumbria (ARC-NENC) and Kent, Surrey and Sussex (ARC-KSS), respectively. The views expressed in this publication are those of the authors and not necessarily those of the NIHR.

For peer review only - http://bmjopen.bmj.com/site/about/guidelines.xhtml

Study Registration: This study is registered with the Open Science Framework (OSF)
https://osf.io/vpjns/?view_only=d2e5c1000a1c4df19d6e478b7e1515de

For peer review only

For peer review only - http://bmjopen.bmj.com/site/about/guidelines.xhtml

References

1 Laing W. Care Homes for Older People: UK Market Report. 30th Edition. London UK: 2019.

2 Gordon AL, Franklin M, Bradshaw L, *et al.* Health status of UK care home residents: a cohort study. *Age Ageing* 2014;**43**:97–103. doi:10.1093/ageing/aft077

3 Alzheimer's Society. Fix Dementia Care: NHS and are homes. London: 2016. https://www.alzheimers.org.uk/download/downloads/id/3026/fix_dementia_care_nhs_and_care_homes_report.pdf (accessed 12 Apr 2018).

4 Burton J, Wolters A, Towers A-M, *et al.* Developing a minimum data set for older adult care homes in the UK: exploring the concept and defining early core principles. *Lancet Heal Longev* 2022;**3**:E186–93.

5 Johnston L, Hockley J, Henderson DAG, *et al.* The development of a care home data platform in Scotland: Insights from the care home innovation partnership, Lothian. *medRxiv* Published Online First: 2020. doi:10.1101/2020.08.17.20176503

6 DHSC. Data saves lives: reshaping health and social care with data (Updated 15 June 2022). *UK Gov* 2022;:1–65.https://www.gov.uk/government/publications/data-saves-lives-reshaping-health-and-social-care-with-data-draft/data-saves-lives-reshaping-health-and-social-care-with-data-draft%0Ahttps://www.gov.uk/government/publications/data-saves-lives-reshaping-health-a

7 Hanratty B, Burton JK, Goodman C, *et al.* Covid-19 and lack of linked datasets for care homes: The pandemic has shed harsh light on the need for a live minimum dataset. BMJ. 2020;**369**. doi:10.1136/bmj.m2463

8 Department of Health and Social Care. People at the Heart of Care: adult social care reform. 2021. https://www.gov.uk/government/publications/people-at-the-heart-of-care-adult-social-care-reform-white-paper/people-at-the-heart-of-care-adult-social-care-reform (accessed 21 Dec 2022).

9 Centers for Medicare and Medicaid Services. Long-Term Care Facility Resident Assessment Instrument 3.0 User's Manual. 2015.

10 Morris JN, Fries BE, Steel K, *et al.* Comprehensive clinical assessment in community setting - applicability of the MDS-HC. *J Am Geriatr Soc* 1997;**45**:1017–24.

11 Carpenter I, Hirdes JP. Using interRAI assessment systems to measure and maintain quality of long-term care. In: *A Good Life in Old Age?: Monitoring and Improving Quality in Long-term Care*. Paris: : OECD Publishing 2013. 93–139. doi:10.1787/9789264194564-7-en

12 Goodman C, Davies SL, Gordon AL, *et al.* Optimal NHS service delivery to care homes: a realist evaluation of the features and mechanisms that support effective working for the continuing care of older people in residential settings. *Heal Serv Deliv Res* 2017;**5**:1–204. doi:10.3310/hsdr05290

13 Stosz L, Carpenter I. Developing the use of MDS/RAI reports for UK care homes. York:

For peer review only - http://bmjopen.bmj.com/site/about/guidelines.xhtml

2008. www.jrf.org.uk (accessed 21 Dec 2022).

14 Goodman C, Steventon A, Killett A, *et al.* Developing research resources And minimum data set for Care Homes ’ Adoption and use (DACHA) study. *NIHR 127234* Published Online First: 2020.https://fundingawards.nihr.ac.uk/award/NIHR127234

15 Peryer G, Kelly S, Blake J, *et al.* Contextual factors influencing complex intervention research processes in care homes: a systematic review and framework synthesis. *Age Ageing* 2022;**51**. doi:10.1093/ageing/afac014

16 Irvine L, Burton JK, Ali M, *et al.* Protocol for the development of a repository of individual participant data from randomised controlled trials conducted in adult care homes (the Virtual International Care Homes Trials Archive (VICHTA)). *Trials* 2021;**22**:1–11. doi:10.1186/S13063-021-05107-W/TABLES/3

17 Musa MK, Akdur G, Brand S, *et al.* The uptake and use of a minimum data set (MDS) for older people living and dying in care homes: a realist review. *BMC Geriatr 2022 221* 2022;**22**:1–14. doi:10.1186/S12877-021-02705-W

18 Wolters A, Santos F, Lloyd T, *et al.* Emergency admissions to hospital from care homes: how often and what for? London: 2019. https://www.health.org.uk/publications/reports/emergency-admissions-to-hospital-from-care-homes

19 Cochran WG. *Sampling Techniques*. 3rd ed. New Tork: : Wiley 1977.

20 Allan S, Forder J. The determinants of care home closure. *Heal Econ (United Kingdom)* 2015;**24**:132–45. doi:10.1002/HEC.3149

21 Devi R, Chadborn NH, Meyer J, *et al.* How quality improvement collaboratives work to improve healthcare in care homes: a realist evaluation. *Age Ageing* 2021;**50**:1371–81. doi:10.1093/AGEING/AFAB007

22 Towers A-M, Smith N, Palmer S, *et al.* The acceptability and feasibility of using the Adult Social Care Outcomes Toolkit (ASCOT) to inform practice in care homes. *BMC Health Serv Res* 2016;**16**:523. doi:10.1186/s12913-016-1763-1

23 Steventon A, Roberts A. Estimating length of stay in publicly-funded residential and nursing care homes: A retrospective analysis using linked administrative data sets. *BMC Health Serv Res* 2012;**12**:377. doi:10.1186/1472-6963-12-377

24 Forder J, Fernandez J-L. Length of stay in care homes. Published Online First: 2011.www.pssru.ac.uk (accessed 12 Apr 2018).

25 Devi R, Meyer J, Banerjee J, *et al.* Quality improvement collaborative aiming for Proactive HEAlthcare of Older People in Care Homes (PEACH): A realist evaluation protocol. *BMJ Open* 2018;**8**:23287. doi:10.1136/bmjopen-2018-023287

26 Logan PA, Horne JC, Allen F, *et al.* A multidomain decision support tool to prevent falls in older people: the FinCH cluster RCT. *Health Technol Assess (Rockv)* 2022;**26**:1–136. doi:10.3310/CWIB0236

27 Mental Capacity Act. Mental Capacity Act. *London Station Off* 2005.

28 NHS Digital. Access data on the Personal Demographics Service - NHS Digital. https://digital.nhs.uk/services/demographics/access-data-on-the-personal-

For peer review only - http://bmjopen.bmj.com/site/about/guidelines.xhtml

demographics-service (accessed 21 Dec 2022).

29 Fenton W, Davison S, Fleming N, *et al.* The State of the adult social care sector and workforce in England. London: 2022. https://www.skillsforcare.org.uk/Adult-Social-Care-Workforce-Data/Workforce-intelligence/documents/State-of-the-adult-social-care-sector/The-state-of-the-adult-social-care-sector-and-workforce-2022.pdf (accessed 21 Dec 2022).

30 Santos F, Conti S, Wolters A. A Novel Method for Identifying Care Home Residents in England: A Validation Study. *Int J Popul Data Sci* 2020;**5**. doi:10.23889/IJPDS.V5I4.1666

31 Damschroder LJ, Aron DC, Keith RE, *et al.* Fostering implementation of health services research findings into practice: A consolidated framework for advancing implementation science. *Implement Sci* 2009;**4**:1–15. doi:10.1186/1748-5908-4-50/TABLES/1

32 QSR International. *NVivo Qualitative Data Analysis Software [Software]. Version 12*. Available from https://qsrinternational.com/nvivo/nvivo-products/: 2019.

33 R Core team. A language and environment for statistical computing. 2021.https://www.r-project.org/

34 StataCorp. Stata Statistical Software: Release 17. 2021.

Twitter: @DACHA_study

For peer review only

For peer review only - http://bmjopen.bmj.com/site/about/guidelines.xhtml

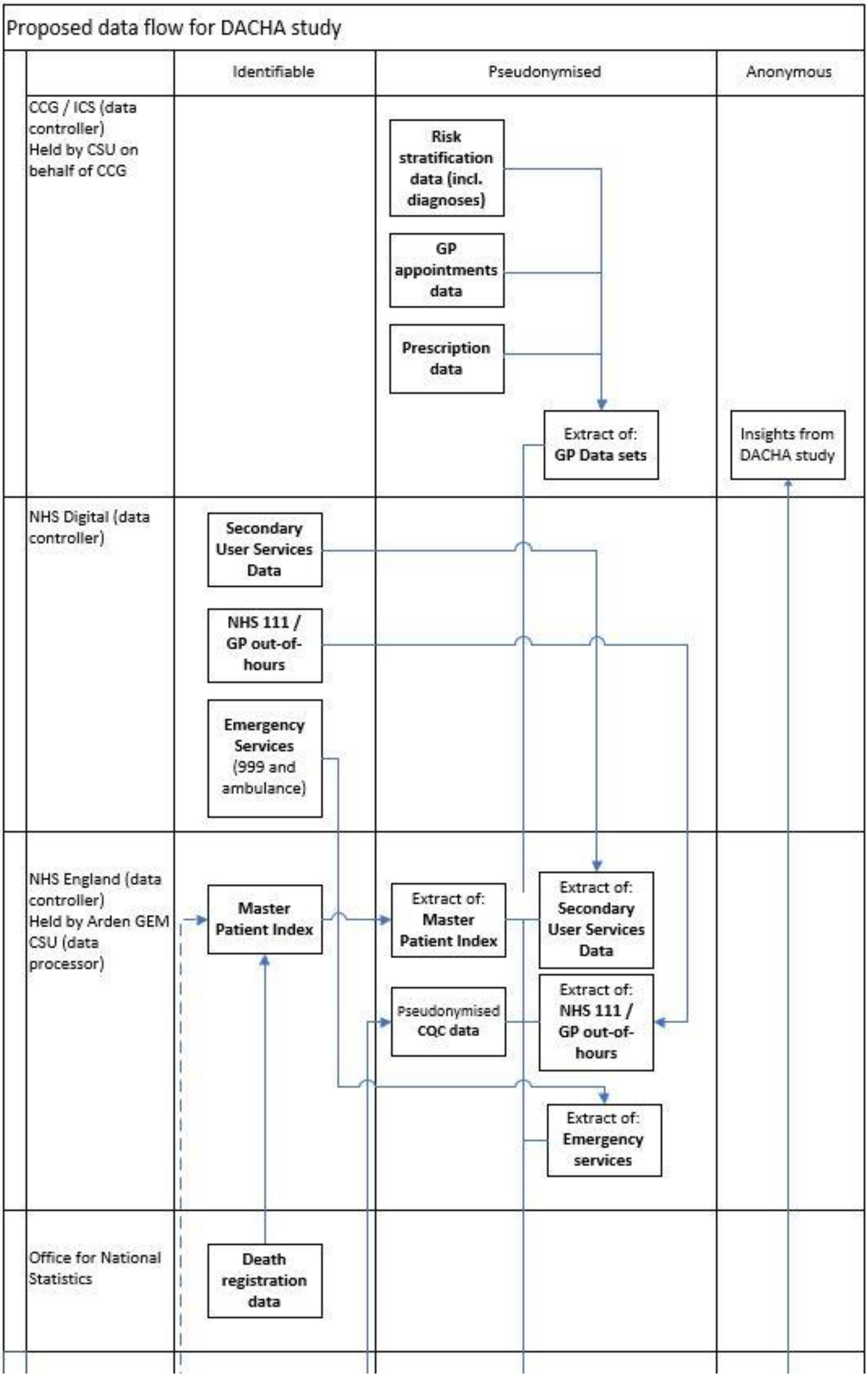
Proposed data flow for DACHA study
Identifiable
Pseudonymised
Anonymous
CCG / ICS (data controller) Held by CSU on behalf of CCG
Risk stratification data (incl. diagnoses)
GP appointments data
Prescription data
Extract of: GP Data sets
Insights from DACHA study
NHS Digital (data controller)
Secondary User Services Data
NHS 111 / GP out-of-hours
Emergency Services (999 and ambulance)
NHS England (data controller) Held by Arden GEM CSU (data processor)
Master Patient Index
Extract of: Master Patient Index
Extract of: Secondary User Services Data
Pseudonymised CQC data
Extract of: NHS 111 / GP out-of-hours
Extract of: Emergency services
Office for National Statistics
Death registration data

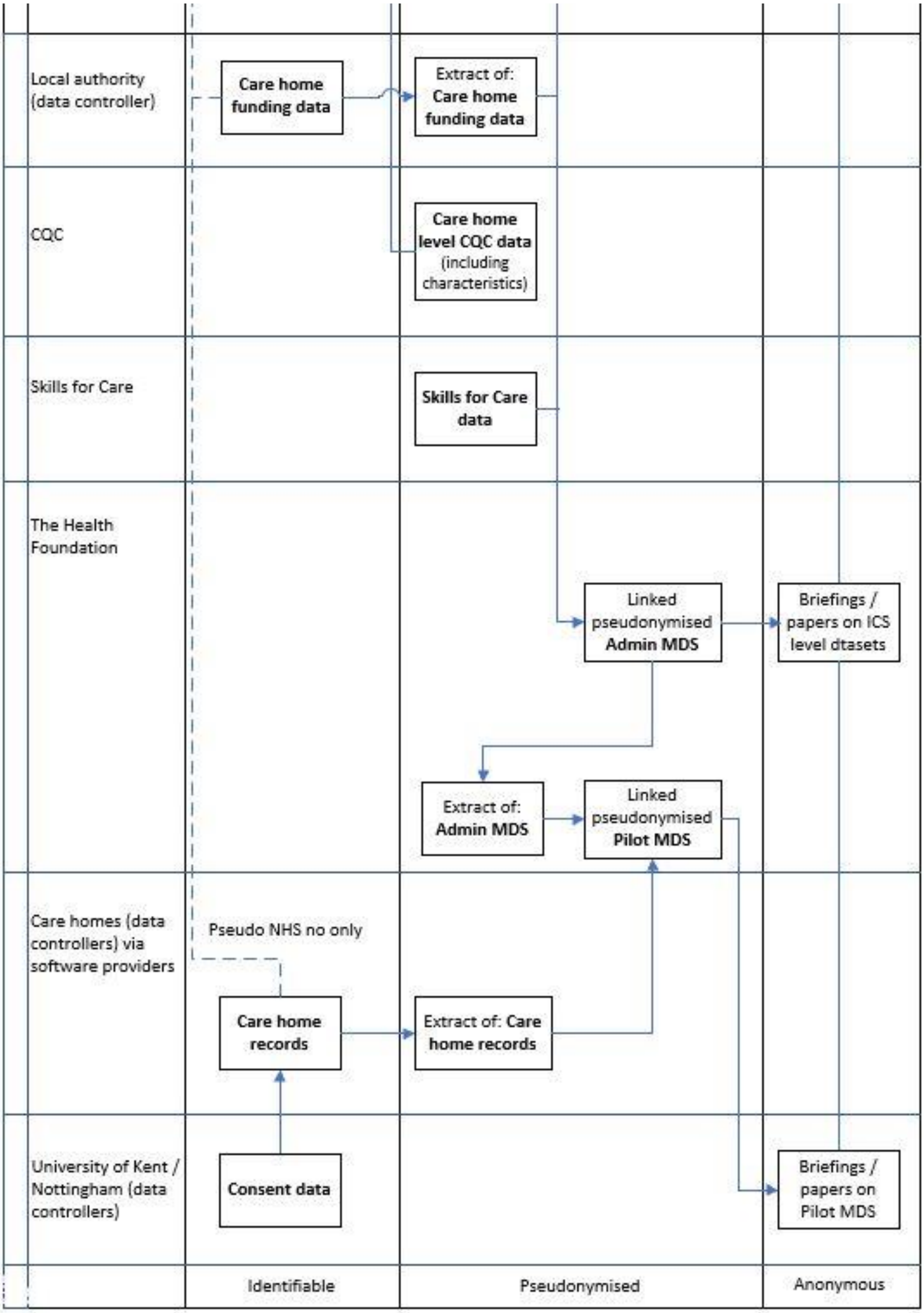
Local authority (data controller)
Care home funding data
Extract of: Care home funding data
CQC
Care home level CQC data (including characteristics)
Skills for Care
Skills for Care data
The Health Foundation
Linked pseudonymised Admin MDS
Briefings / papers on ICS level dtasets
Extract of: Admin MDS
Linked pseudonymised Pilot MDS
Care homes (data controllers) via software providers
Pseudo NHS no only
Care home records
Extract of: Care home records
University of Kent / Nottingham (data controllers)
Consent data
Briefings / papers on Pilot MDS
Identifiable
Pseudonymised
Anonymous

BMJ Open

Piloting of a minimum data set for older people living in care homes in England: protocol for a longitudinal, mixed-methods study

Journal:	*BMJ Open*
Manuscript ID	bmjopen-2023-071686.R1
Article Type:	Protocol
Date Submitted by the Author:	06-Feb-2023
Complete List of Authors:	Towers, Ann-Marie; University of Kent, Centre for Health Services Studies; NIHR Applied Research Collaboration Kent, Surrey and Sussex<br>Gordon, Adam; University of Nottingham, School of Medicine; NIHR Applied Research Collaboration East Midlands<br>Wolters, Arne; The Health Foundation, Data Analytics<br>Allan, Stephen; University of Kent, Personal Social Services Research Unit<br>Rand, Stacey; University of Kent, Personal Social Services Research Unit<br>Webster, Lucy; University of Kent, Centre for Health Services Studies<br>Crellin, Elizabeth; The Health Foundation, Improvement Analytics Unit<br>Brine, Richard; The Health Foundation, Data Analytics<br>De Corte, Kaat; The Health Foundation, Data Analytics<br>Akdur, Gizdem; University of Hertfordshire, Centre for Research in Public health and Community Care<br>Irvine, Lisa; University of Hertfordshire, Centre for Research in Public health and Community Care (CRIPACC)<br>Burton, Jennifer ; University of Glasgow, Institute of Cardiovascular and Medical Sciences<br>Hanratty, Barbara; University of Newcastle upon Tyne, Institute of Health and Society / Newcastle University Institute for Ageing; NIHR Applied Research Collaboration North East and North Cumbria<br>Killett, Anne; University of East Anglia, School of Health Sciences; NIHR Applied Research Collaboration East of England<br>Meyer, Julienne; City University, School of Health Sciences; National Care Forum<br>Jones, Liz; National Care Forum<br>Goodman, Claire; University of Hertfordshire, Centre for Research in Public Health and Community Care; NIHR Applied Research Collaboration East of England
**Primary Subject Heading**:	Health services research
Secondary Subject Heading:	Geriatric medicine
Keywords:	Protocols & guidelines < HEALTH SERVICES ADMINISTRATION & MANAGEMENT, Quality in health care < HEALTH SERVICES ADMINISTRATION & MANAGEMENT, GERIATRIC MEDICINE

Piloting of a minimum data set for older people living in care homes in England: protocol for a longitudinal, mixed-methods study

Abstract

Introduction

Health and care data is routinely collected about care home residents in England, yet there is no way to collate these data to inform benchmarking and improvement. The Developing research resources And minimum data set for Care Homes' Adoption and use (DACHA) study has developed a prototype minimum data set (MDS) for piloting.

Methods and analysis

A mixed-methods longitudinal pilot study will be conducted in 60 care homes (approximately 960 residents) in three regions of England, using resident data from cloud-based digital care home records at two-time points. These will be linked to resident and care home level data held within routine NHS and social care data sets. Two rounds of focus groups with care home staff (n=8-10 per region) and additional interviews with external stakeholders (n=3 per region) will explore implementation and the perceived utility of the MDS. Data will be assessed for completeness and timeliness of completion. Descriptive statistics, including percentage floor and ceiling effects will establish data quality. For validated scales, construct validity will be assessed by hypothesis testing and exploratory factor analysis will establish structural validity. Internal consistency will be established using Cronbach's alpha. Longitudinal analysis of the pilot data will demonstrate the value of the MDS to each region. Qualitative data will be analysed inductively using thematic analysis to understand the complexities of implementing an MDS in care homes for older people.

Ethics and dissemination

The study has received ethical approval from the London Queen's Square Research Ethics Committee [22/LO/0250]. Informed consent is required for participation. Findings will be disseminated to: academics working on data use and integration in social care, care sector organisations, policy makers, and commissioners. Findings will be published in peer-reviewed journals. Partner NIHR Applied Research Collaborations, the National Care Forum, and the British Geriatrics Society will disseminate policy briefs.

Strengths and limitations of this study

- Matching digital care record data with data held about residents in routine datasets addresses the need for better integrated health and care data highlighted during the COVID-19 pandemic.

For peer review only - http://bmjopen.bmj.com/site/about/guidelines.xhtml

- We cannot guarantee the linked data will be nationally representative, but we will explore and report on the typicality of participating homes, using national data.
- To mitigate against the risk of data not being matched due to recording errors in residents' NHS numbers, we will verify NHS numbers in the digital care home records and measure the extent to which it occurs.

Introduction

Approximately 420,000 older people live in care homes in England and Wales. Just under half of residents are admitted to care homes following hospital admission [1]. Older care home residents live with high levels of dependency, cognitive impairment and multi-morbidity [2]. Around 70% have dementia, though some will not have a formal diagnosis [3].

Data are routinely collected and held about residents by care homes and external agencies (e.g. NHS, LAs, the regulator). Some of this information is returned to different regulators, commissioners and policymakers for aggregated use. The lack of harmonisation of indicators means duplication of effort because similar information about residents is recorded in different ways for different uses [4]. Heterogeneity in the frequency of measurement, tools and measures deployed and the mode of recording [5], makes it difficult to compare or collate data at an aggregate level. Recent estimates suggest that approximately 30-40% of social care providers in England are still entirely paper-based, with another third only partially digital [6].

The pandemic highlighted the need to collate real-time information about residents' health and care needs and accelerated the move to digital data solutions through emergency legislation [6,7]. Tools, such as the Capacity Tracker in England, originally designed to manage hospital discharges, were repurposed to monitor infection and vaccination uptake rates, and collect workforce data. The new white paper, People at the Heart of Care, aims to reverse historical under-investment in adult social care technology [8]. The Government target is for 80% of care providers (including care homes) to be using digital care records by March 2024 [6]. As well as improving the accessibility and interoperability of routinely collected data in care homes, this presents opportunities to standardise the way data is recorded about residents' needs and outcomes for the purposes of an MDS.

Structured and standardised approaches to data in long-term care are well established in some countries. The Minimum Data Set (MDS 3.0) [9] in the United States is mandated for all nursing home residents funded by Medicare or Medicaid. The International Resident Assessment Instrument (interRAI) [10], initially derived from the MDS, is used in multiple countries including Canada and New Zealand, France, Belgium, Netherlands, Australia and Switzerland. These datasets enable care coordination, governance and audit, remuneration for services, service design and planning, and policy decisions [11]. Previous efforts to introduce interRAI in the UK have been unsuccessful [12,13], possibly because data capture

For peer review only - http://bmjopen.bmj.com/site/about/guidelines.xhtml

was not incorporated into routine practice and was perceived as an additional administrative burden [4].

The DACHA study [14] is a programme of research (2019-2024) that aims to develop and test an MDS for care homes providing care to older people in England. There are five work packages (WPs), three of which are complete: systematic evidence reviews to inform measures and outcomes for inclusion in an MDS (WP1) [15]; creation of an individual patient data repository of UK care home trials (WP2) [16]; and realist review identifying programme theories describing what is involved when developing and implementing an MDS in care homes (WP3) [17].

Adopting an approach used in previous work [18], WP4, led by the Health Foundation, will produce a resident-level MDS from data *routinely collected* by health and social care providers. These data will not come directly from care homes, although homes will have populated some of it for other purposes (e.g. for the Care Quality Commission (CQC)). Care home residents will be identified based on the address registered with their General Practitioner [18]. This protocol is for WP5, in which we augment WP4 data, to form a complete MDS, using data extracted from care homes' digital care records.

Methods and analysis

Aim and objectives

Aim

To undertake a longitudinal pilot of a prototype MDS for older people living in care homes using digital care records in England.

Objectives

1. Assess the feasibility of extracting data directly from digital care records and matching this to routinely collected health and social care data to populate a complete MDS.
2. Assess the quality of the complete MDS data.
3. Evaluate the usefulness of the MDS to stakeholders (health and social care, local authorities, care providers, residents and their families).
4. Assess potential barriers and facilitators to wider implementation of the MDS.

Study design

A mixed-methods longitudinal pilot of the prototype MDS will be conducted in 60 care homes for older people (nursing and residential) using digital care records from participating

For peer review only - http://bmjopen.bmj.com/site/about/guidelines.xhtml

software vendors in three sites (n=20 homes per site)[1] and linking this data to routinely held health and social care data (see Table 1 for data sources). Each site will comprise an Integrated Care System (ICS). An ICS is a regional partnership between NHS organisations, local councils and other organisations including the third sector and social enterprises, to coordinate and provide health and social care. There are 42 ICSs across England. The study has chosen three ICSs (Nottingham and Nottinghamshire, Surrey Heartlands, and North East and North Cumbria), with a history of working collaboratively with care homes to improve care delivery.

Using a longitudinal design will enable us to assess the feasibility of the MDS by looking at completion rates and implementation over time.

The prototype MDS

Given government policy to move rapidly to digital shared care records [6], we will work with homes that already have digital care software to plan and record residents' care. The final MDS, consisting of routinely-held health and social care data and data extracted from care records, will include information about residents' demographics, date of admission, health and care needs (including palliative care), quality of life, complications and adverse events, diagnoses, medication, vaccinations, health care use, care home characteristics, and workforce data (see Table 1 for data sources).

We are working with the Care Software Providers Association (CASPA), a consortium of software providers (*https://caspa.care*), and two software companies (Person Centred Software (*https://personcentredsoftware.com/gb/*) and Nourish (*https://nourishcare.co.uk/*). We have mapped the DACHA MDS variables against the data routinely collected in their systems and are working with them to address any gaps. For example, previous work identified that different stakeholders wanted information about residents' quality of life (QoL), yet this is not currently routinely collected [4,17]. QoL measures are therefore being added to the software for the pilot and will be available to care homes to inform direct care. We are also working with providers to add other items (e.g. cognitive impairment and activities of daily living) in a standardised format. Currently, descriptive care notes capture important information for direct care (e.g. about functioning) but not in a format suitable for quantitative analysis.

Sampling

Homes

We aim to recruit 20 care homes for older people in three ICSs (total N=60 homes) geographically dispersed across England (South East, Midlands and North East). The three

[1] Care homes for people with intellectual and developmental disabilities will not be included in this study.

For peer review only - http://bmjopen.bmj.com/site/about/guidelines.xhtml

ICSs have 279 care homes (11,121 beds), 222 homes (8,451 beds), and 636 homes (26,968 beds) respectively. Assuming an occupancy rate of 90% [1], the sample size required to give a true representation of the finite older care home population in each of the three ICSs, with 90% confidence and a 5% margin of error, is 262-268 residents [19].

Care homes will be eligible for inclusion if they are routinely using digital care records from the two participating software vendors. Based on figures provided by the software vendors, the eligible sample across the three respective sites for this study are 52 homes (3,093 beds), 51 homes (2,809 beds), and 66 homes (3,288 beds). To achieve an adequate resident sample for the study, we will stratify the eligible care homes by size, maintaining an even split of homes with and without on-site nursing (nursing and residential homes) in each ICS, where possible. We anticipate that approximately three out of every four care homes selected will be above average size (40 beds).

Based on earlier work and cognisant of ongoing post pandemic pressures we assume a conservative 33% response rate for residents [20] . Given the stratifying of eligible care homes, anticipated response rate and assuming 90% occupancy this would provide a sample of 358, 320 and 292 residents in each of the three ICSs on average (n=970 residents in total). Median length of stay for residents in care homes will vary by location and provider [18,21,22]. We will liaise with care home managers to assess any potential issue with occupancy rates and sample attrition (particularly recognising the smaller sample size in ICS three), looking to identify ways to resolve this, for example by setting higher recruitment targets for specific homes.

Residents

Inclusion and exclusion criteria

We will utilise a census approach where all permanent residents will be eligible to participate, including those lacking capacity to consent. To minimise research burden on potential participants going through acute transitions, and who may also move through the care home rapidly, we will exclude residents receiving respite or temporary/short-stay care, or people identified by the care home staff as being in the last weeks of life at the point of study inception.

Recruitment

All residents' capacity to consent will be assessed by a research nurse or member of the research team. We will support the recruitment of adults without mental capacity by using a personal consultee for those with a relative to act on their behalf, and a professional or nominated consultee for residents with nobody to act for them. We have used this approach in previous studies [12,23,24], in line with the Mental Capacity Act [25]. Consultee discussions may take place on the telephone or using videoconferencing technology.

For peer review only - http://bmjopen.bmj.com/site/about/guidelines.xhtml

Data collection from care homes

Resident data collected from care homes will be entirely extracted from the home's digital care records via the participating software vendors at baseline and six months. Participating care homes will be the data controllers. The software providers will be the data processors, meaning they will pseudonymise the data for participating residents (see data linkage below for details) and support the homes to transfer it securely to The Health Foundation. The pseudonymised data will be stored securely on The Health Foundation servers.

Data linkage and processing

Table 1 summarises the data to be collected and proposed data sources. We will use deterministic linkage to merge the data from digital care records with the routinely collected health and social care administrative data to complete the prototype MDS (see, Data Flow Diagram in Supplementary information). This will include data on: demographics and care home stay [source – Personal Demographics Service] [26]; adverse events, diagnoses, medications and health care utilisation (e.g. primary care and hospital appointments, ambulance call outs) [sources - GP, Secondary Uses Service, NHS111 data sets]; care home characteristics [sources – CQC, local authority data sets]; and basic workforce data [source – Skills for Care] [27]. We will identify relevant participant identifiers, for example NHS number and/or National Insurance number, to complete this linkage. These identifiers will subsequently be pseudonymised using an agreed linkage key, allowing the data from digital care records to be linked to administrative data pseudonymised in the same way [28].

The linked dataset will be held by The Health Foundation in a dedicated secure data environment. The pseudonymisation key will be shared separately with the other data providers supplying administrative data for the purpose of the MDS. The Health Foundation staff will not have access to the pseudonymisation key but instead link the datasets together using pseudonymised identifiers, therefore making it unlikely that resident data can be re-identified.

All resident information will be processed in a dedicated secure environment that complies with national data security standards (NHS Digital Data Protection and Security Toolkit) and certified against an international standard for information security (ISO27001), access to this environment will be limited to trained analysts, and all analysis findings are subjected to rigorous statistical disclosure control procedures to make sure residents cannot be identified.

Care home level data

We will collect data on institutional variables for each care home to contextualise the evaluation and consider representativeness of the wider care home sector. To minimise

For peer review only - http://bmjopen.bmj.com/site/about/guidelines.xhtml

research burden for care homes much of this data will be collected from the routinely held health and social care data described above. Data collected directly from care homes through a short online survey will include: number of beds; number of residents; proportion of self-funding residents; and the number of full-time equivalent care staff currently employed.

For peer review only

Table 1. Proposed data sources for the prototype MDS

	Sections	Example variables	Digital care records	Health and social care datasets
1	Demographics/ characteristics	Date of birth; sex; NHS number; area-based deprivation	No	Personal Demographics Service
		Religion, Languages, Marital or partnership status, Deprivation of liberty	Yes	No
		Ethnicity; weight; height	Yes	GP data; Secondary User Services data
2	Palliative Care Needs	End of life pathway register	No	GP data
3	Care Home Stay	Date of entry to care home; date of death	Yes	No
4	Resident Needs	Skin condition	Yes	No
		Cognitive impairment and impact on perception, understanding & need for support	Yes*	GP data; Secondary User Services data
		Oral/nutritional status	No	Secondary User Services data
		Continence	No	Community datasets (where available)
		Ability to perform activities of daily living; cognitive performance; delirium	Yes*	No
5	Quality of Life	Outcomes; mood; dementia quality of life	Yes*	No
6	Complications / Adverse Events	Infections	Yes	GP data; Secondary User Services data
		Falls (leading to hospital admission or GP visit)	Yes	Secondary User Services data; 999 data; Ambulance data

For peer review only - http://bmjopen.bmj.com/site/about/guidelines.xhtml

	Sections	Example variables	Digital care records	Health and social care datasets
		Falls (only captured at care home level); early warning score; unintended weight loss	Yes	No
7	Diagnoses	Past medical history	No	Secondary User Services data; GP data
		Frailty	No	GP data
		Adverse reactions and allergies	No	GP data
8	Medication and Vaccination	Prescribed medication and administered vaccines	No	GP data
9	Health Care Utilisation	Primary care use	No	GP data; NHS 111 data; 999 data
		Community nursing; Community allied health professionals	No	Community Services Data Set
		Out-of-hours contacts	No	GP out-of-hours data
		Ambulance call outs	No	Ambulance data
		A&E attendance; emergency admissions; secondary care usage (outpatient appointments & elective admission)	No	Secondary User Services data
10	Care Home Characteristics and workforce characteristics	Type of home; care home characteristics, specialities and client groups; location of care home; area-based deprivation; registered bed capacity; sector of provider; provider ownership type; CQC rating	No	CQC data
		Staffing model; staffing ratios; numbers and types of staff; number of agency staff; number and type of vacancies	No**	Skills for Care data

*Will be added to the software for the purposes of the pilot study

**As the Skills for Care workforce survey is voluntary, we will ask participating homes to provide some information on workforce as part of a short online survey for the pilot.

For peer review only - http://bmjopen.bmj.com/site/about/guidelines.xhtml

For peer review only

Feedback to care homes

We will give participating homes aggregated summaries of their own MDS data, benchmarked against other data from the whole sample in their ICS. This will include indicators of residents' health, use of services and social care outcomes (e.g. quality of life). We seek to understand the extent to which the MDS may generate useful information for care homes, esepcially in relation to service development and improvement.

Support for homes

To provide appropriate support to homes throughout, each ICS will have a dedicated, local researcher who will be their main point of contact for the study duration. Homes will be offered payment of £100 per home, per wave of data extraction (£200 in total), as a token of appreciation. Where possible, measures used in the pilot will be questionnaires developed for and/or used in care homes already and designed to be completed by staff. This should improve implementation and reduce burden on staff [15].

Focus groups and interviews

Care home managers and staff will be invited to participate in two rounds of focus group interviews (up to two hours long) with other care homes from their ICS or one-to-one interviews (up to an hour long) (see Table 2). These focus groups and interviews will explore:

- Implementation issues;
- Applicability and utility of the MDS for direct care;
- Potential modifications to the MDS.

Participation in these focus groups and interviews is voluntary, informed consent will be obtained and participants thanked for their time with a £20 gift voucher. All will be audio-recorded, with interviews facilitated by one researcher, and focus groups facilitated by two researchers. We will keep focus groups to a manageable size (8-10 per group), running additional groups to accommodate views of staff from all participating homes if required.

At the end of the pilot, we will conduct telephone or face-to-face interviews with three key representatives from each ICS (n=9) to explore views of the implementation process and relevance of the data to their local priorities. To assess potential barriers and facilitators to the wider implementation of the MDS, the implementation aspects of the interviews and focus groups will be structured using the Consolidated Framework for Implementation Research (CFIR) [29].

A final stakeholder event will be held in each participating ICS area to present summary data on the MDS, constituent variables and linked health and social care outcomes. Group discussions will explore utility and feasibility of the MDS.

For peer review only - http://bmjopen.bmj.com/site/about/guidelines.xhtml

Table 2. Research activities by wave of data collection

	Data extraction and linkage	Aggregated feedback to homes	Focus groups and interviews
Wave 1	March 2023	May 2023	May-June 2023
Wave 2	September 2023	November 2023	November-Dec 2023

For peer review only

Data synthesis and analysis

This study will extract resident-level data from digital care records and pilot the process of matching this to routinely held health and social care data sources, collated by The Health Foundation. In addition, there will be data from focus groups and interviews. As such, there will be both quantitative and qualitative data collected by the study at different levels (resident, care home and ICS). We will use NVivo for windows [30] to store and analyse qualitative data. We will use R [31] and STATA [32] to analyse quantitative data.

Analysis of the implementation process

Analysis of the implementation process will be structured to test findings of earlier DACHA work describing what enables uptake and use of MDS in care home settings and what is perceived by different stakeholders as key to successful uptake (for example, shared aims, utility, ease of use) [15,17]. Data from each ICS will be analysed separately, to identify locality themes and then together to identify common themes.

Considering the psychometrics of the MDS

Descriptive statistics will be reported for MDS data, including the percentage floor (lowest score) and ceiling (highest score) for multi-item measures. Feasibility will be assessed by completeness (percentage missing data). To identify factors related to completion rates, bivariate analysis of the % completion rates for the MDS variables added for the pilot (e.g. QoL measures) and care home organisational factors (e.g. CQC rating, care home size, staffing level) will be conducted. For validated measures (e.g. quality of life), exploratory factor analysis will be used to assess the structural validity of the data in the MDS. Construct (convergent) validity will be assessed by hypothesis testing where there are either multiple measures of similar constructs, for example around Quality of Life, or we would expect to observe associations based on previously published research. Internal consistency of participants' responses will be described using Cronbach's alpha.

For peer review only - http://bmjopen.bmj.com/site/about/guidelines.xhtml

Analysing the ways in which MDS can be useful to key stakeholders

We will demonstrate the value of the MDS to stakeholders through analysis of the pilot data. Alongside The Health Foundation, we will introduce the prototype MDS to representatives of each ICS and seek to understand their local geography, service delivery and commissioning structures. Priority research and service development questions will be identified that can be addressed using the pilot data in an appropriate longitudinal study. For example, the MDS could be used to assess what drives hospital admissions from care homes. Sample size and power calculations will be considered during these discussions.

We have worked hard, through preparatory work in earlier work packages, to produce a parsimonious dataset that will minimise data collection burden on care homes. We will, though, at this point also explore variables, missing from the current MDS, which may improve its usefulness to key stakeholders.

Patient and public involvement

The DACHA study includes a programme of patient and public involvement (PPI) which holds regular meetings of patient and public involvement groups with relatives of people living in care homes, and care home staff in both direct care and management roles. Leadership of this group includes someone with experience both as a family carer and working in a care home. Care home residents are involved through collaboration with activity providers in their care homes. Stakeholders, including care providers, commissioners, quality monitoring and health providers are involved through structured national consultations. These stakeholders have shaped the earlier work packages, which have then fed into this MDS pilot. PPI representatives also sit on the study steering committee and attend team meetings.

For this pilot study, involvement has included consultation with PPI representatives from care homes, family carers and residents regarding the consent process, information sheets and consent forms, priorities for content of an MDS, quality of life measures included in the MDS, principles of data sharing and pseudonymization.

Input of PPI representatives will be sought throughout the study, commenting on findings from interviews and focus groups, and measurement issues raised by individual data items, or groups of items, within the dataset. We will also work with this group to develop Plain English dissemination materials.

Ethics and dissemination

The study has received a favourable ethical approval from the London – Queen's Square Research Ethics Committee [22/LO/0250]. Residents and staff will all receive written and verbal information about the study from the research team or research nurses supporting the study. Where participants have capacity, informed consent will be gained. In accordance

For peer review only - http://bmjopen.bmj.com/site/about/guidelines.xhtml

with the Mental Capacity Act [25], residents assessed as lacking the capacity to consent will be recruited via the advice of a personal or nominated consultee.

Findings will be disseminated to three key audiences: academics working on data use and integration in long term care, care sector representative organisations, and health and social care policy makers and commissioners. This will be through presentations at academic and clinical and sector specific conferences, webinars tailored for stakeholder groups, a report to the funder and publications in peer-reviewed journals. Findings will be summarised in plain English briefings and social media publications accessible to residents, relatives, and care home staff. We will work with our partner NIHR Applied Research Collaborations, the National Care Forum colleagues, and the British Geriatrics Society, the national professional organisation representing health care professionals with an interest in the care of older people with frailty, to develop policy briefs aimed at commissioners and providers of health and social care.

The end product of this research will be a finalised digital MDS prototype. Although this will have been tested in English care homes, our aim is to develop a tool that will be useful across all four UK nations, with international relevance. This work will also create new knowledge on how to support care homes and other stakeholders to implement the prototype MDS, as well as the data linkage arrangements required. Ultimately, this will enable us to make recommendations that encourage widespread use of the MDS [4,6]. Piloting the MDS with homes already using digital care records will help to future-proof its use, and inform the Department of Health and Social Care's agenda for digitalisation of health and social care records [6].

This study is registered with the Open Science Framework (OSF; https://osf.io/vpjns/?view_only=d2e5c1000a1c4df19d6e478b7e1515de).

Discussion

The care home sector is heterogeneous, and this is a pilot of an MDS in care homes using digital record software providers. This approach is in line with policy directives for 80% of providers to be using digital care records by March 2024. We are working with two software providers with the largest market coverage in England. Recruiting homes in three different regions of England, including one site with high social deprivation, will also maximise the likelihood our cohort will sample a range of geographical and socio-demographic contexts. The sampling strategy described above aims to recruit a mixture of homes that are representative of the sector in terms of type (residential and nursing care), proprietorial status and size. Whilst we cannot ensure that the linked data will be nationally representative, we will be able to explore and report on the typicality of participating homes using national data held by the Health Foundation in WP4

All permanent residents will be eligible (except those known to be in the last weeks of life). This will enable us to attain a representative sample of care home residents for each ICS.

For peer review only - http://bmjopen.bmj.com/site/about/guidelines.xhtml

Sampling could be affected by local variation in occupancy rates and length of stay, but we will be able to detect such variation using our data [18].

Our data linkage approach bringing together care home generated data with resident data held in routine datasets is innovative and addresses the need for integrated data. There are some risks. First, the NHS number used to link records could be recorded incorrectly, meaning the pseudo key will not match. We can mitigate against it by verifying NHS number in digital care home records and measuring the extent to which it occurs. Second, residents consented in the pilot are not captured by the administrative dataset because their registered address has not been updated or is not recognised as a care home by the Unique Property Reference Number (UPRN) algorithms used by The Health Foundation. We will mitigate against this by sharing the NHS Number directly from the providers to the NHS.

The dataset will be deployed in a real-world setting and then describe how organisational and individual stakeholder factors impact implementation using mixed methods. We will be able to understand the feasibility and usefulness of the dataset, refine its contents and establish how to implement it more widely at scale and pace. This work takes place against a backdrop of ongoing post pandemic service reconfiguration. We are mitigating against this by working with organisations active around data in care homes, including government agencies, to ensure our findings are cognisant of, and compatible with, other work underway or planned.

This pilot will demonstrate the use and value of an MDS in care homes already using digital care planning software that is linked to resident data held routinely by local health and social care organisations. The rapid acceleration towards digital shared care records and the imminent establishment of a data framework for adult social care, underline the need for empirical evidence about what needs to be in place to implement these changes, whilst ensuring data are relevant to those providing, funding and regulating care for care home residents.

*** ***

Supplementary appendix

Data Flow Diagram: This is a flow diagram of the data being collected in the pilot study for the purposes of the MDS. It shows data sources and linkage.

Contributors

The study concept and design was conceived by CG, AMT, AG, AW, BH, JB, LI and LJ. SA, SR, RB, EC, KDC, LW and GA assisted in refining the design and creating tables and figures. AW, SA and SR designed the analysis plan, with input from the team. JM and AK led on PPI input. AMT led the drafting of the manuscript, with input from AG, AW, LW, SR and SA. All authors critically revised the manuscript and approve the submitted version.

Competing interests

None declared.

For peer review only - http://bmjopen.bmj.com/site/about/guidelines.xhtml

Funding

This work is funded by National Institute for Health Research (NIHR) Health Services and Delivery Research (HS&DR) Programme, grant number NIHR127234. Adam Gordon, Claire Goodman, Anne Killett, Barbara Hanratty and Ann-Marie Towers are part-funded by the NIHR Applied Research Collaborations for East Midlands (ARC-EM), East of England (ARC-EoE), North East and North Cumbria (ARC-NENC) and Kent, Surrey and Sussex (ARC-KSS), respectively. The views expressed in this publication are those of the authors and not necessarily those of the NIHR.

Study registration

This study is registered with the Open Science Framework (OSF) https://osf.io/vpjns/?view_only=d2e5c1000a1c4df19d6e478b7e1515de

Twitter: @DACHA_study

For peer review only

References

1 Laing W. Care Homes for Older People: UK Market Report. 30th Edition. London UK: 2019.

2 Gordon AL, Franklin M, Bradshaw L, *et al.* Health status of UK care home residents: a cohort study. *Age Ageing* 2014;**43**:97–103. doi:10.1093/ageing/aft077

3 Alzheimer's Society. Fix Dementia Care: NHS and are homes. London: 2016. https://www.alzheimers.org.uk/download/downloads/id/3026/fix_dementia_care_nhs_and_care_homes_report.pdf (accessed 12 Apr 2018).

4 Burton J, Wolters A, Towers A-M, *et al.* Developing a minimum data set for older adult care homes in the UK: exploring the concept and defining early core principles. *Lancet Heal Longev* 2022;**3**:E186–93.

5 Johnston L, Hockley J, Henderson DAG, *et al.* The development of a care home data platform in Scotland: Insights from the care home innovation partnership, Lothian. *medRxiv* Published Online First: 2020. doi:10.1101/2020.08.17.20176503

6 DHSC. Data saves lives: reshaping health and social care with data (Updated 15 June 2022). *UK Gov* 2022;:1–65.https://www.gov.uk/government/publications/data-saves-lives-reshaping-health-and-social-care-with-data-draft/data-saves-lives-reshaping-health-and-social-care-with-data-draft%0Ahttps://www.gov.uk/government/publications/data-saves-lives-reshaping-health-a

7 Hanratty B, Burton JK, Goodman C, *et al.* Covid-19 and lack of linked datasets for care homes: The pandemic has shed harsh light on the need for a live minimum dataset. BMJ. 2020;**369**. doi:10.1136/bmj.m2463

8 Department of Health and Social Care. People at the Heart of Care: adult social care reform. 2021. https://www.gov.uk/government/publications/people-at-the-heart-of-care-adult-social-care-reform-white-paper/people-at-the-heart-of-care-adult-social-care-reform (accessed 21 Dec 2022).

For peer review only - http://bmjopen.bmj.com/site/about/guidelines.xhtml

9 Centers for Medicare and Medicaid Services. Long-Term Care Facility Resident Assessment Instrument 3.0 User's Manual. 2015.

10 Morris JN, Fries BE, Steel K, *et al.* Comprehensive clinical assessment in community setting - applicability of the MDS-HC. *J Am Geriatr Soc* 1997;**45**:1017–24.

11 Carpenter I, Hirdes JP. Using interRAI assessment systems to measure and maintain quality of long-term care. In: *A Good Life in Old Age?: Monitoring and Improving Quality in Long-term Care*. Paris: : OECD Publishing 2013. 93–139. doi:10.1787/9789264194564-7-en

12 Goodman C, Davies SL, Gordon AL, *et al.* Optimal NHS service delivery to care homes: a realist evaluation of the features and mechanisms that support effective working for the continuing care of older people in residential settings. *Heal Serv Deliv Res* 2017;**5**:1–204. doi:10.3310/hsdr05290

13 Stosz L, Carpenter I. Developing the use of MDS/RAI reports for UK care homes. York: 2008. www.jrf.org.uk (accessed 21 Dec 2022).

14 Goodman C, Steventon A, Killett A, *et al.* Developing research resources And minimum data set for Care Homes ' Adoption and use (DACHA) study. *NIHR 127234* Published Online First: 2020.https://fundingawards.nihr.ac.uk/award/NIHR127234

15 Peryer G, Kelly S, Blake J, *et al.* Contextual factors influencing complex intervention research processes in care homes: a systematic review and framework synthesis. *Age Ageing* 2022;**51**. doi:10.1093/ageing/afac014

16 Irvine L, Burton JK, Ali M, *et al.* Protocol for the development of a repository of individual participant data from randomised controlled trials conducted in adult care homes (the Virtual International Care Homes Trials Archive (VICHTA)). *Trials* 2021;**22**:1–11. doi:10.1186/S13063-021-05107-W/TABLES/3

17 Musa MK, Akdur G, Brand S, *et al.* The uptake and use of a minimum data set (MDS) for older people living and dying in care homes: a realist review. *BMC Geriatr 2022 221* 2022;**22**:1–14. doi:10.1186/S12877-021-02705-W

18 Wolters A, Santos F, Lloyd T, *et al.* Emergency admissions to hospital from care homes: how often and what for? London: 2019. https://www.health.org.uk/publications/reports/emergency-admissions-to-hospital-from-care-homes

19 Cochran WG. *Sampling Techniques*. 3rd ed. New Tork: : Wiley 1977.

20 Towers A-M, Smith N, Palmer S, *et al.* The acceptability and feasibility of using the Adult Social Care Outcomes Toolkit (ASCOT) to inform practice in care homes. *BMC Health Serv Res* 2016;**16**:523. doi:10.1186/s12913-016-1763-1

21 Steventon A, Roberts A. Estimating length of stay in publicly-funded residential and nursing care homes: A retrospective analysis using linked administrative data sets. *BMC Health Serv Res* 2012;**12**:377. doi:10.1186/1472-6963-12-377

22 Forder J, Fernandez J-L. Length of stay in care homes. Published Online First: 2011.www.pssru.ac.uk (accessed 12 Apr 2018).

23 Devi R, Meyer J, Banerjee J, *et al.* Quality improvement collaborative aiming for

For peer review only - http://bmjopen.bmj.com/site/about/guidelines.xhtml

Proactive HEAlthcare of Older People in Care Homes (PEACH): A realist evaluation protocol. *BMJ Open* 2018;**8**:23287. doi:10.1136/bmjopen-2018-023287

24 Logan PA, Horne JC, Allen F, *et al.* A multidomain decision support tool to prevent falls in older people: the FinCH cluster RCT. *Health Technol Assess (Rockv)* 2022;**26**:1–136. doi:10.3310/CWIB0236

25 Mental Capacity Act. Mental Capacity Act. *London Station Off* 2005.

26 NHS Digital. Access data on the Personal Demographics Service - NHS Digital. https://digital.nhs.uk/services/demographics/access-data-on-the-personal-demographics-service (accessed 21 Dec 2022).

27 Fenton W, Davison S, Fleming N, *et al.* The State of the adult social care sector and workforce in England. London: 2022. https://www.skillsforcare.org.uk/Adult-Social-Care-Workforce-Data/Workforce-intelligence/documents/State-of-the-adult-social-care-sector/The-state-of-the-adult-social-care-sector-and-workforce-2022.pdf (accessed 21 Dec 2022).

28 Santos F, Conti S, Wolters A. A Novel Method for Identifying Care Home Residents in England: A Validation Study. *Int J Popul Data Sci* 2020;**5**. doi:10.23889/IJPDS.V5I4.1666

29 Damschroder LJ, Aron DC, Keith RE, *et al.* Fostering implementation of health services research findings into practice: A consolidated framework for advancing implementation science. *Implement Sci* 2009;**4**:1–15. doi:10.1186/1748-5908-4-50/TABLES/1

30 QSR International. *NVivo Qualitative Data Analysis Software [Software]. Version 12*. Available from https://qsrinternational.com/nvivo/nvivo-products/: 2019.

31 R Core team. A language and environment for statistical computing. 2021.https://www.r-project.org/

32 StataCorp. Stata Statistical Software: Release 17. 2021.

For peer review only - http://bmjopen.bmj.com/site/about/guidelines.xhtml

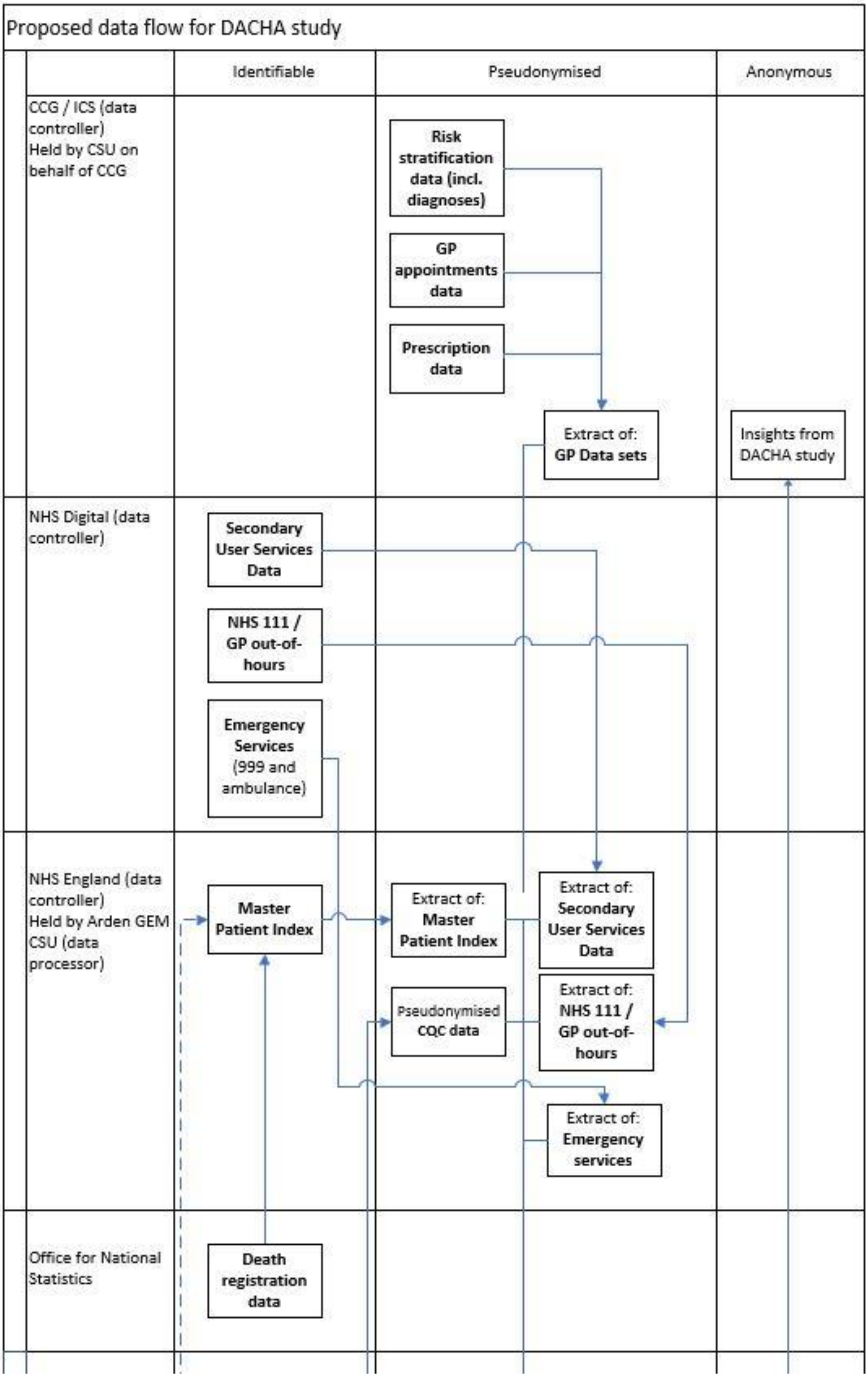
Proposed data flow for DACHA study
Identifiable
Pseudonymised
Anonymous
CCG / ICS (data controller) Held by CSU on behalf of CCG
Risk stratification data (incl. diagnoses)
GP appointments data
Prescription data
Extract of: GP Data sets
Insights from DACHA study
NHS Digital (data controller)
Secondary User Services Data
NHS 111 / GP out-of-hours
Emergency Services (999 and ambulance)
NHS England (data controller) Held by Arden GEM CSU (data processor)
Master Patient Index
Extract of: Master Patient Index
Extract of: Secondary User Services Data
Pseudonymised CQC data
Extract of: NHS 111 / GP out-of-hours
Extract of: Emergency services
Office for National Statistics
Death registration data

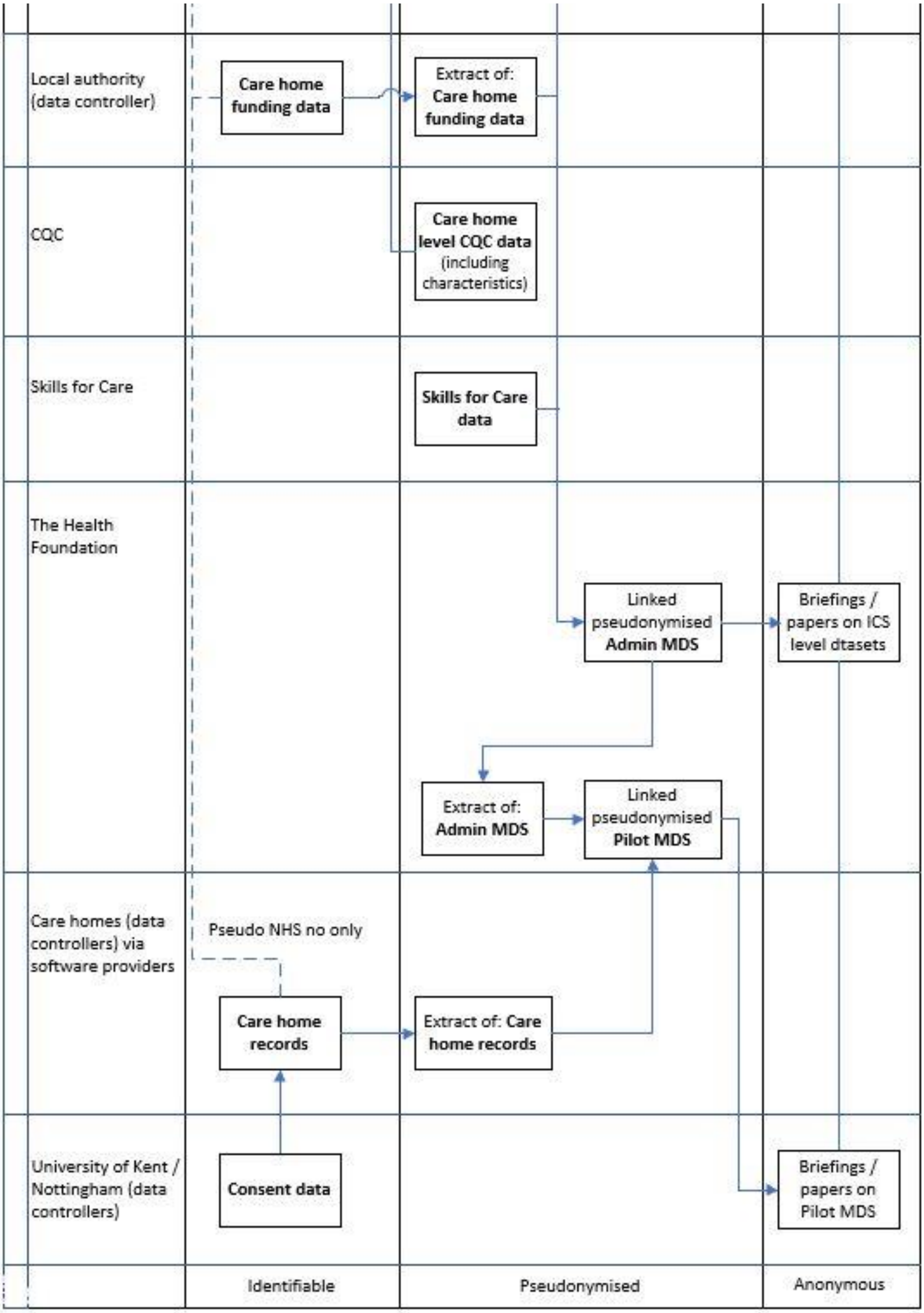
Local authority (data controller)
Care home funding data
Extract of: Care home funding data
CQC
Care home level CQC data (including characteristics)
Skills for Care
Skills for Care data
The Health Foundation
Linked pseudonymised Admin MDS
Briefings / papers on ICS level dtasets
Extract of: Admin MDS
Linked pseudonymised Pilot MDS
Care homes (data controllers) via software providers
Pseudo NHS no only
Care home records
Extract of: Care home records
University of Kent / Nottingham (data controllers)
Consent data
Briefings / papers on Pilot MDS
Identifiable
Pseudonymised
Anonymous
