# Supplementary material for: Piloting of a minimum data set for older people living in care homes in England: protocol for a longitudinal, mixed-methods study
Source: BMJ Open. 2023 Feb 27;13(2):e071686. doi: 10.1136/bmjopen-2023-071686 (PMC9972423; doi:10.1136/bmjopen-2023-071686)
Supplement: Reviewer comments [file bmjopen-2023-071686.reviewer_comments.pdf]

PEER REVIEW HISTORY

BMJ Open publishes all reviews undertaken for accepted manuscripts. Reviewers are asked to complete a checklist review form (**http://bmjopen.bmj.com/site/about/resources/checklist.pdf**) and are provided with free text boxes to elaborate on their assessment. These free text comments are reproduced below.

ARTICLE DETAILS

**TITLE (PROVISIONAL)**	Piloting of a minimum data set for older people living in care homes in England: protocol for a longitudinal, mixed-methods study
**AUTHORS**	Towers, Ann-Marie; Gordon, Adam; Wolters, Arne; Allan, Stephen; Rand, Stacey; Webster, Lucy; Crellin, Elizabeth; Brine, Richard; De Corte, Kaat; Akdur, Gizdem; Irvine, Lisa; Burton, Jennifer; Hanratty, Barbara; Killett, Anne; Meyer, Julienne; Jones, Liz; Goodman, Claire

This article was not externally reviewed at BMJ Open. Protocols that have been independently assessed prior to submission to BMJ Open are usually fast-tracked to publication on the grounds that further substantial changes will not be possible. This independent assessment will usually be external, independent review for both a substantial grant award from a non-commercial or government funder and ethics approval.
